# Research on a Wind Turbine Gearbox Fault Diagnosis Method Using Singular Value Decomposition and Graph Fourier Transform

**DOI:** 10.3390/s24103234

**Published:** 2024-05-20

**Authors:** Lan Chen, Xiangfeng Zhang, Zhanxiang Li, Hong Jiang

**Affiliations:** College of Intelligent Manufacturing and Industrial Modernization, Xinjiang University, Urumchi 830017, China; 107556521223@stu.xju.edu.cn (L.C.); 107552204222@stu.xju.edu.cn (Z.L.); onlyxjjh@xju.edu.cn (H.J.)

**Keywords:** singular value decomposition, graph Fourier transform, gearbox, fault diagnosis

## Abstract

Gearboxes operate in challenging environments, which leads to a heightened incidence of failures, and ambient noise further compromises the accuracy of fault diagnosis. To address this issue, we introduce a fault diagnosis method that employs singular value decomposition (SVD) and graph Fourier transform (GFT). Singular values, commonly employed in feature extraction and fault diagnosis, effectively encapsulate various fault states of mechanical equipment. However, prior methods neglect the inter-relationships among singular values, resulting in the loss of subtle fault information concealed within. To precisely and effectively extract subtle fault information from gear vibration signals, this study incorporates graph signal processing (GSP) technology. Following SVD of the original vibration signal, the method constructs a graph signal using singular values as inputs, enabling the capture of topological relationships among these values and the extraction of concealed fault information. Subsequently, the graph signal undergoes a transformation via GFT, facilitating the extraction of fault features from the graph spectral domain. Ultimately, by assessing the Mahalanobis distance between training and testing samples, distinct defect states are discerned and diagnosed. Experimental results on bearing and gear faults demonstrate that the proposed method exhibits enhanced robustness to noise, enabling accurate and effective diagnosis of gearbox faults in environments with substantial noise.

## 1. Introduction

Gearboxes, known for their smooth transmission and reliable operation, are extensively employed in the primary drive chains of wind turbines [1]. However, owing to the harsh operational environment and the cumulative effects of multiple excitation sources, gearbox failures are inevitable, compromising the overall functionality of the equipment and potentially leading to severe safety incidents [2]. Statistical data indicate that bearings and gears frequently fail during gearbox operation, necessitating wind turbine downtime for maintenance [3]. Therefore, monitoring the operational status of these components is crucial.

However, constrained by the operational environment, the collected vibration signals are frequently embedded in a noisy background, complicating fault diagnosis and reducing fault identification performance in practical applications. The singular value decomposition (SVD) algorithm, known for its heightened sensitivity to weak signals amidst noisy backgrounds, is frequently utilized to extract subtle fault information [4,5]. Diverse singular values within the sequence characterize different signal components and serve directly as fault features [6], prompting numerous scholars to employ singular values in depicting equipment fault states. For instance, Yanfeng Li et al. [7] utilized the entire sequence of singular values from vibration signals as fault features, employing deep neural networks as classifiers to identify rolling bearing faults. Building on this, Zhang Lizhi et al. [8] and Wang Fengtao et al. [9] initially decomposed the original vibration signal into multiple intrinsic mode functions (IMFs) using the empirical mode decomposition (EMD) algorithm. Subsequently, they selected appropriate IMFs to construct, respectively, the time–frequency domain’s spatial state matrix or Hankel matrix, extracting the singular values of these matrices as feature vectors for diagnosis via deep neural networks. Similarly, Zhong et al. [10] constructed the vibration signal into a state matrix by employing the enhanced empirical mode decomposition (EEMD) algorithm, utilizing the matrix’s singular values as feature vectors.

The aforementioned methods construct the state matrix that describes the vibration signal in various ways and utilize the singular values of this matrix to characterize the operating state of the mechanical equipment. However, while these approaches emphasize developing a more comprehensive state matrix, they often overlook the inter-relationships among the singular values, thereby failing to extract holistic fault information from the vibration signal. To address this issue, this paper introduces the graph signal processing (GSP) technique, an extension of discrete signal processing theory within the domain of graph-structured signal processing. This approach offers a novel perspective for processing vibration signals and has been extensively applied in fault diagnosis by numerous scholars. For instance, Lu et al. [11] constructed the original signal into a graph model and detected changes in equipment operational status from the resultant graph signal. Gao et al. [12] utilized the total variance of the graph signal as the characteristic indicator of bearing faults by transforming the bearing’s vibration signal into a graphically interpretable form. Wang Hao et al. [13] and Ou et al. [14] transformed the vibration signal into a roadmap and introduced, respectively, a GFT-based impact feature extraction method utilizing optimal weighting and a rolling bearing fault diagnosis method leveraging GFT pulse component extraction. These studies pioneer a novel research direction by applying GSP technology, transitioning fault diagnosis from traditional time and frequency domain processing to vertex and map domain processing, thereby enhancing application performance. Liao X et al. [15] introduces a novel fault diagnosis method for power grids based on the Graph Fourier Transform (GFT). The method leverages GFT to analyze the grid’s topological structure and operational data, enabling more accurate and efficient fault detection and diagnosis.

Drawing upon the preceding discussion, this study introduces a fault diagnosis methodology for gearboxes that leverages SVD and GFT, incorporating GSP technology. The method capitalizes on the sensitivity of singular values to detect faults, constructs graph signals using these values as inputs, and explores the topological relationships among the values via the graph structure. This approach facilitates the extraction of obscured fault information, enabling robust fault diagnosis amidst substantial noise interference. Experimental validations on bearing and gear faults demonstrate that the methodology delineated in this paper effectively accomplishes gearbox fault diagnosis. Moreover, it exhibits greater stability and reliability under noise disturbances compared to conventional methods.

## 2. Theoretical Basis of the Study

### 2.1. Singular Value Decomposition

SVD theory, an inherent characteristic of matrices, demonstrates that singular values exhibit minimal changes and maintain high stability when the matrix is perturbed. Consequently, the principal singular values of a vibration signal exhibit negligible variations before and after noise interference. For any real matrix, it can be decomposed into singular vectors and singular values through a transformation involving the multiplication of orthogonal matrices to both its left and right sides. In this paper, the Hankel matrix is used to construct the vibration signal as an eigenmatrix, and for a matrix *H* ∈ *R^m^*^×^*^n^*, there exist orthogonal matrices *U* and *V* such that the following equation holds:

(1) $$H=\left[\begin{array}{cccc} x_{1} & x_{2} & \cdots & x_{n}\\ x_{2} & x_{3} & \cdots & x_{n+1}\\ \vdots & \vdots & \ddots & \vdots \\ x_{m} & x_{m+1} & \cdots & x_{N} \end{array}\right]=U_{m\times m}S_{m\times n}V_{n\times n}^{T}$$

where *x* is the vibration signal; *H* is the Hankel matrix constructed using the vibration signal; and the matrix *S* satisfies

(2) $$S_{m\times n}=\left[\begin{array}{cc} S_{r\times r} & 0\\ 0 & 0 \end{array}\right]$$

where *S_r_*_×_*_r_* = diag (σ_1_, σ_2_, …, σ*_r_*), where σ*_i_*= (1, 2, …, *r*) is the singular value of the matrix *H* and *r* = min(*m*, *n*). Equation (1) is the singular value decomposition process for any *m* × *n* matrix.

### 2.2. Graph Signal Processing Techniques

#### 2.2.1. Visibility Algorithm

**Figure 1:**
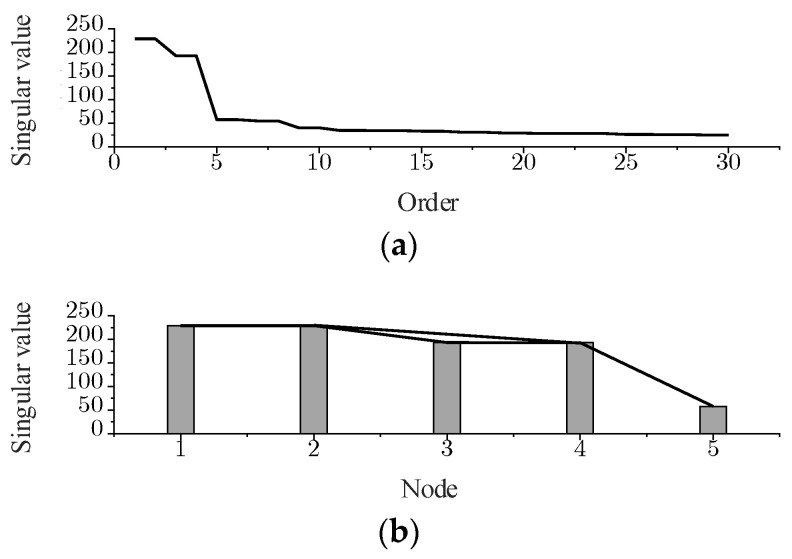
Visibility graph of singular value sequence. (**a**) Singular value sequence. (**b**) Network of singular value sequence.

The Visibility Graph (VG) algorithm [16,17], a method for transforming a finite-length discrete time series into a complex network, ensures that each network node corresponds to an individual element of the time series. The fundamental concept of this method is illustrated in Figure 1. In this study, a sequence of singular values serves as the input for the visibility algorithm. To prevent the schematic from becoming overly cluttered, only the first five singular values are demonstrated. In Figure 1b, the singular values inputted into the visibility algorithm are depicted as histogram bars, with the height of each bar corresponding to the magnitude of the singular values.

If the tops of two histograms satisfy visibility, the corresponding two vertices are considered to be connected by an edge. The visibility criterion is defined as follows: if any 2 points, (*t_a_*, *y_a_*) and (*t_b_*, *y_b_*), in the input sequence have visibility, then these two points become two connected nodes in the graph. Then for any point, (*t_c_*, *y_c_*), where *t_a_* < *t_c_* < *t_b_*, satisfies

(3) $$y_{c}<y_{b}+(y_{a}-y_{b})\frac{t_{b}-t_{c}}{t_{b}-t_{a}}$$

The visibility algorithm delineates the relationships between nodes and edges within the network by defining specific connection rules among nodes. Subsequently, it transforms the discrete signal into a graph signal based on these rules. Moreover, it employs the Laplacian matrix to characterize the topology of the input sequence. Then, for the viewable signal in Figure 1b, the set of vertices is denoted as $V=\{v_{1},v_{2},v_{3},v_{4},v_{5}\}$, the set of edges is denoted as $E=\{(v_{1},v_{2}),(v_{2},v_{3}),(v_{3},v_{4}),(v_{4},v_{5}),(v_{2},v_{4})\}$, and the adjacency matrix is denoted as

$$W=\left(\begin{array}{ccccc} 0 & 1 & 0 & 0 & 0\\ 1 & 0 & 1 & 1 & 0\\ 0 & 1 & 0 & 1 & 0\\ 0 & 1 & 1 & 0 & 1\\ 0 & 0 & 0 & 1 & 0 \end{array}\right)$$

The degree diagonal matrix is expressed as

$$D=\left(\begin{array}{ccccc} 1 & 0 & 0 & 0 & 0\\ 0 & 3 & 0 & 0 & 0\\ 0 & 0 & 2 & 0 & 0\\ 0 & 0 & 0 & 3 & 0\\ 0 & 0 & 0 & 0 & 1 \end{array}\right)$$

The Laplace matrix is expressed as

$$L=D-W=\left(\begin{array}{ccccc} 1 & -1 & 0 & 0 & 0\\ -1 & 3 & -1 & -1 & 0\\ 0 & -1 & 2 & -1 & 0\\ 0 & -1 & -1 & 3 & -1\\ 0 & 0 & 0 & -1 & 1 \end{array}\right)$$

#### 2.2.2. Graph Fourier Transform

A graph signal is a mapping defined on the set of graph vertices, denoted by a vector $f\in R^{N}$, where the *i*th component of the vector corresponds to the value of the first vertex in the set of vertices *V*, which, in this paper, is the first singular value in the sequence of singular values. The GFT [18] is an extension of the Fourier transform (FT) to the graph signal, which extends the FT to the graph. Similar to the definition of FT, GFT performs projection based on the eigenvectors of the Laplace matrix, with the difference that the matrix used in FT has a fixed form and the basis function remains unchanged, while the matrix used in GFT varies with the graph structure and the eigenvectors change with the way the weights in the adjacency matrix are defined. GFT can be applied to analyze any undirected, connected, weighted graph, and the GFT result $\hat{f}(r)$ for the graph signal f(n) is expressed by the definition as

(4) $$\hat{f}(r)=\sum_{n=1}^{N}u_{r}(n)f(n)$$

Then, the inversion of GFT is defined as

(5) $$f(n)=\sum_{r=0}^{N-1}\hat{f}(r)u_{r}(n)$$

where r∈{0,1,⋯,N−1} is the order of the eigenvalues and eigenvectors, and $u_{r}$ is the eigenvector of the Laplacian matrix *L*.

The graph Fourier transform (GFT) decomposes a graph signal into a superposition of eigenvectors, each associated with distinct eigenvalues, thereby establishing a correspondence between the graph signal and its eigenvalue spectrum [13]. Similarly to how the Fourier transform (FT) converts a vibration signal from the time domain to the frequency domain, the GFT transforms a graph signal from the vertex domain to the eigenvalue spectrum within the spectral domain, accentuating the data hidden within the vertex domain in the spectral domain.

## 3. Simulated Signal Analysis

The interdependencies among singular values can be leveraged to extract higher-order fault information for diagnostics [19], a consideration often overlooked by traditional methods. The graph structure inherently extracts correlations between data elements by incorporating connections among edges in node pairs. Furthermore, graph signal processing (GSP) theory links the data’s topology with the data themselves via this graph structure [20]. Thus, constructing visibility signals with singular values fully utilizes the graph topology to elucidate relationships among these values, thereby effectively extracting fault information embedded in their interdependencies to diagnose various faults.

To validate the stability of the singular value visibility signal within a noisy environment, we examined a noisy harmonic signal composed of amplitude-modulated frequency components, cosine components, and noise elements, as delineated in Equation (6):

(6) $$\left\{\begin{array}{l} y(t)=y_{1}(t)+y_{2}(t)+\mathrm{noise}\\ y_{1}(t)=2\sin(20\pi t)\\ y_{2}(t)=4(1+\sin(5\pi t))\cos(50\pi t) \end{array}\right.$$

where ‘noise’ denotes random noise.

**Figure 2:**
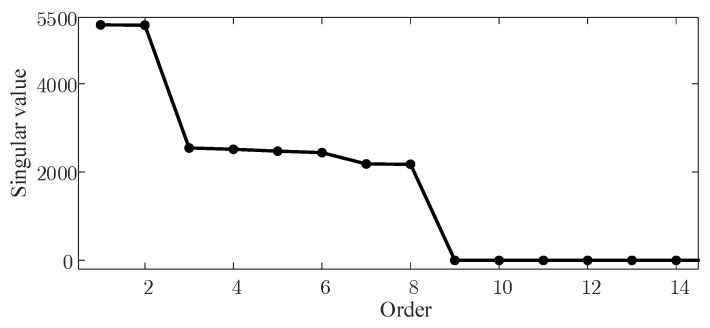
Graph spectra domain of simulated signal.

**Figure 3:**
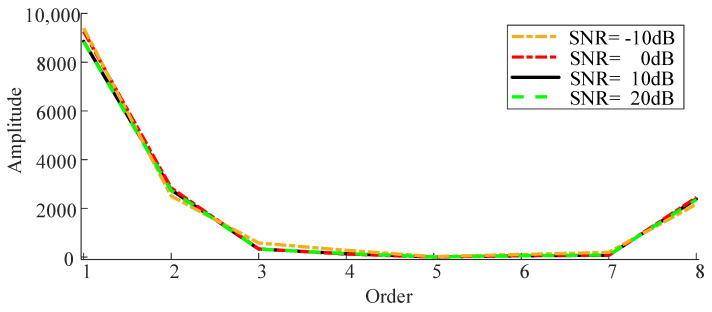
Graph spectra domain of simulated signal.

In the experimental setup, the sampling frequency was established at 5000 Hz, with a sampling duration of 1 s. Initially, the simulated signal was constructed into a Hankel matrix, which was subsequently subjected to singular value decomposition (SVD). Given that the sequence of singular values is arranged in descending order, with the initial values significantly larger than those that follow, these predominant values generally represent the primary components of the vibration signal, while the remaining values correspond to noise. Therefore, the first eight singular values were identified as the main components in the simulated signal, as shown in Figure 2, and subsequently, the visibility signal underwent a graph Fourier transform (GFT). As the first eight singular values are the principal components of the simulated signal, only these were utilized as the input for the Visibility Graph (VG) algorithm, before the visibility signal was finally processed using GFT. The spectral domain of the simulated signal was plotted under varying signal-to-noise ratios of −10 dB, 0 dB, 10 dB, and 20 dB, with the corresponding results displayed in Figure 3.

An examination of Figure 3 reveals that both the amplitude and the overall fluctuations in the spectral domain of the singular value visibility signal under varying signal-to-noise ratios exhibit minimal changes and show substantial overlap, suggesting that the signal spectra are minimally impacted by noise.

Consequently, singular values serve to characterize the intrinsic components of the vibration signal. The interdependencies among these values are discerned through the edge connection rules applied within the visibility algorithm, with the singular values as inputs. Additionally, the spectrum of the singular value visibility signal exhibits high stability and minimal noise influence. Therefore, the energy magnitude and fluctuation degree of the spectrum are utilized as characteristic parameters for fault diagnosis in environments with substantial noise. Furthermore, the standard deviation and energy of the spectrum are designated as characteristic parameters, as defined by Equation (7) in this study.

(7) $$T=\frac{STD\times E}{STD+E}$$

where *STD* is the standard deviation; *E* is the energy.

When the vibration signal exhibits a periodic shock component due to a local fault or an amplitude increase stemming from a distributed fault, the product of the energy standard deviation of the eigenvalue spectrum of the singular value visibility signal is notably larger. Conversely, when the vibration signal changes smoothly, the product of the energy standard deviation of the eigenvalue spectrum is correspondingly smaller; therefore, this study employs the product of energy standard deviation to quantify the abrupt changes in the eigenvalue spectrum, serving as a characteristic parameter for gear fault diagnosis.

## 4. Fault Diagnosis Method and Experimental Verification

### 4.1. Fault Diagnosis Method Based on SVD and GFT

**Figure 4:**
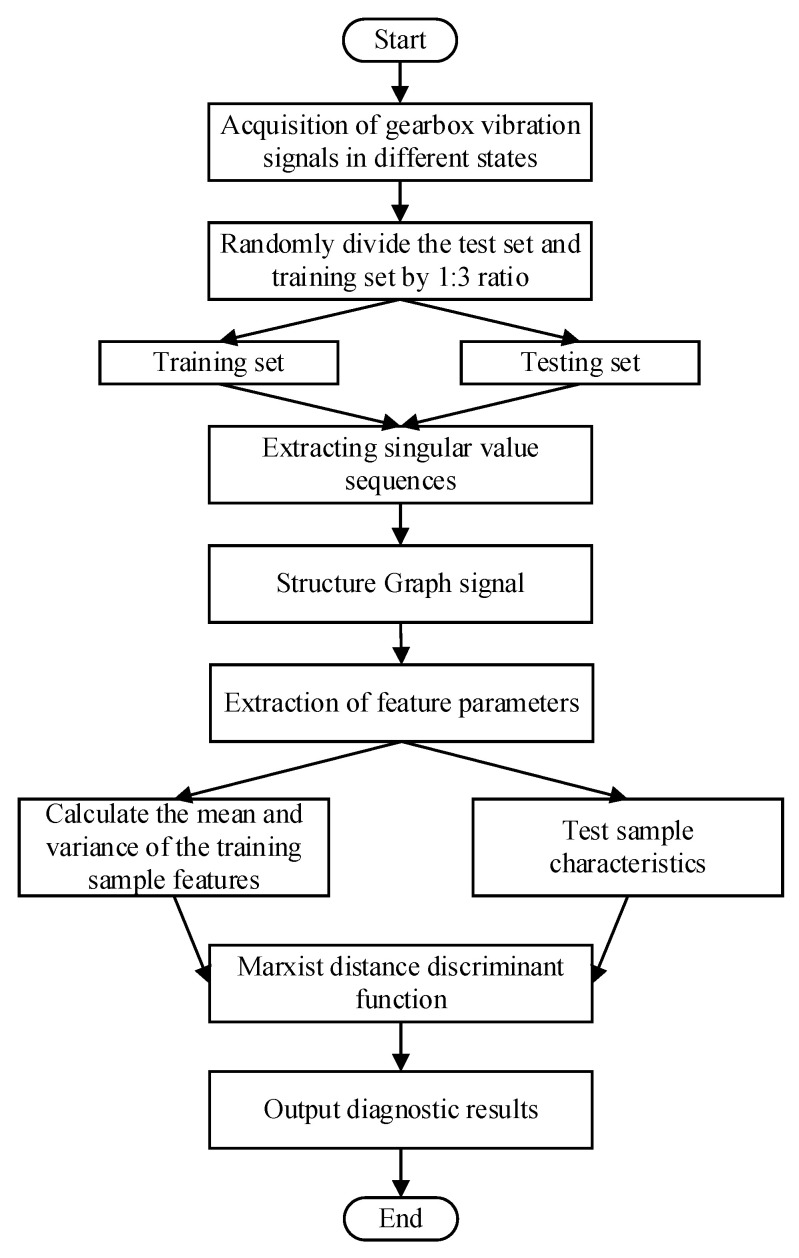
Algorithm flow chart.

Building on the discussion above, the fault diagnosis methodology proposed in this paper is illustrated in Figure 4 and outlined as follows:

Step 1. Randomly select n from the original sample set as test samples and the rest as training samples.

Step 2. Perform SVD on each sample, select the first k singular values as the input of the viewable algorithm, and construct the viewable signal f(k).

Step 3. Perform GFT on the viewable signal f(k), transform it to the spectral domain, calculate the energy and standard deviation, respectively, and synthesize them by Equation (6) as the fault characteristic parameters of the corresponding samples.

Step 4: Calculate the martingale distances between the fault characteristic parameters of each test sample and those of the training samples for each state in sequence.

Step 5: Identify the test samples corresponding to the first n minimal values among each set of distances. Determine the fault types for these test samples based on the state categories of the corresponding training samples, thereby completing the fault identification process.

### 4.2. Experimental Data Description

**Figure 5:**
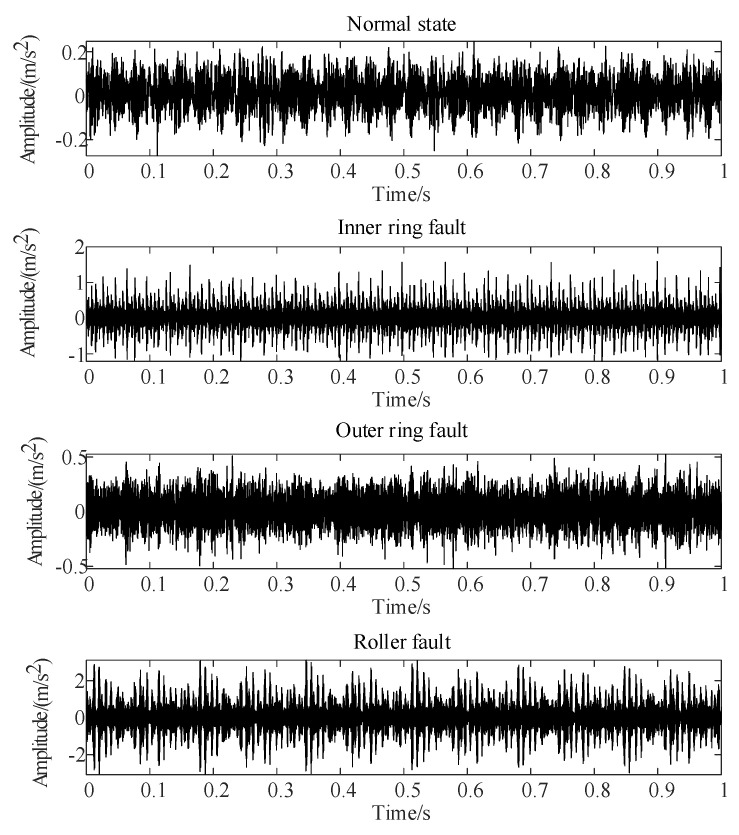
Vibration signals of bearing under different conditions.

To evaluate the effectiveness of the proposed method, tests were conducted using fault signals from both bearings and gears. The bearing fault data originated from the rolling bearing test dataset provided by Case Western Reserve University [21]. Specifically, the tests involved a 6203-2RS JEM SKF deep groove ball bearing under a load of 735.5 W, at a speed of 1797 rpm, with fault dimensions of 0.1778 mm in diameter and 0.2794 mm in depth, and a sampling frequency of 12 kHz. The dataset included various conditions such as the normal state, inner ring failure, outer ring failure, and rolling element failure. Vibration signals from these conditions are shown in Figure 5.

**Figure 6:**
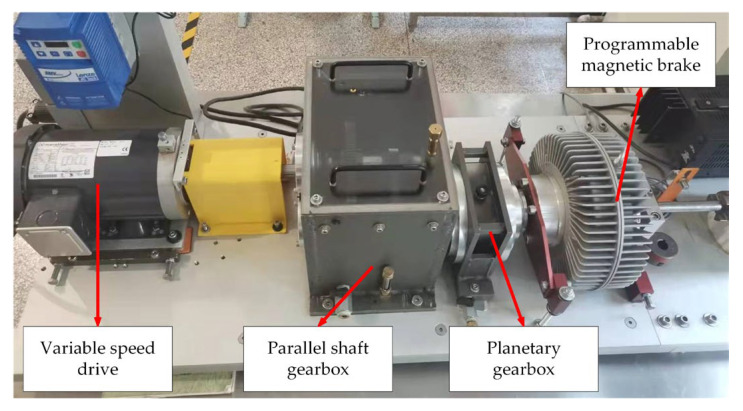
Wind turbine drive system fault simulation test bench.

**Figure 7:**
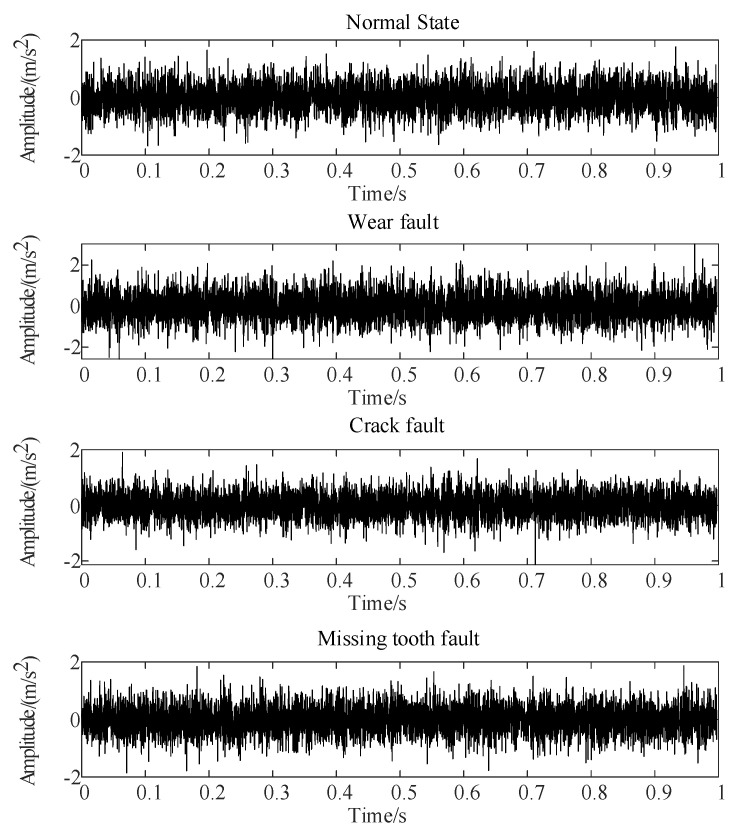
Vibration signals of gear under different conditions.

Gear failure experiments were conducted using a WTDS test stand provided by Spectra Quest (Richmond, VA, USA). This setup included an electric motor, a speed controller, a parallel shaft gearbox, a planetary gearbox, and a magnetic powder brake, as illustrated in Figure 6. During the experiments, all faulty gears were first-stage active wheels with artificially created faults. The data were captured at a theoretical input shaft speed of 1000 rpm, with a sampling frequency of 20.48 kHz. The resulting vibration signals, displayed in Figure 7, demonstrate the impact of noise, which obscures the periodic shock components usually visible in time-domain diagrams, with minimal variation in signal amplitude.

## 5. Experiments and Analyses

### 5.1. Experimental Results and Analysis

**Figure 8:**
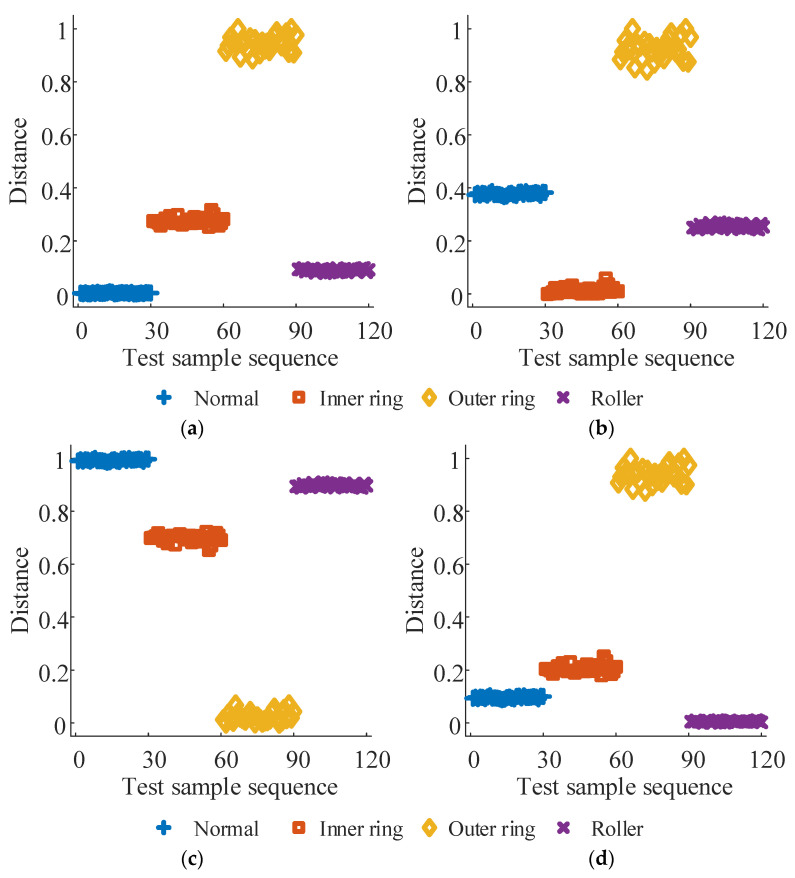
Bearing fault diagnosis results. (**a**) MD1, (**b**) MD2, (**c**) MD3, and (**d**) MD4.

For each bearing vibration signal state, 40 samples were collected, with 10 designated as training samples and the remainder as test samples, resulting in 40 training and 120 test samples. The test sample sequence is organized sequentially: normal state samples (1–30), inner ring failure samples (31–60), outer ring failure samples (61–90), and rolling element failure samples (91–120). Following the methodology outlined in this paper, the training and test samples undergo separate processing. Firstly, the vibration signal undergoes singular value decomposition (SVD), selecting the first nine maxima from the singular value sequence to construct the visibility signal. Subsequently, the Laplace matrix is computed for graph Fourier transform (GFT). Following this, characteristic parameters are computed for each sample using Equation (6). Finally, the Marxian distance is employed to measure the similarity between test and training samples. The diagnostic results are presented in Figure 8. In Figure 8, subplots (a) to (d) represent the Marxian distances (MD1 to MD4) between the test sample sequence and the training samples for the normal state, inner ring fault, outer ring fault, and rolling body fault, respectively. Each subplot of Figure 8 exhibits noticeable distinctions between test samples of different states, with minimal distances observed between samples of the same fault state. This suggests that the fault diagnosis method proposed in this paper is adept at distinguishing between different bearing faults.

Following step 5, the first 30 test samples with the smallest distances in each subplot are identified and deemed to share the same fault state as the training samples. Subsequently, in Figure 8a, the martingale distances between the test samples in sequences 1–30 and the training samples for the normal state correspond to the first 30 minimum values. Thus, the state category is identified as the normal state. Similarly, in Figure 8b–d, the test samples in sequences 31–60, 61–90, and 91–120 are classified as inner ring faults, outer ring faults, and rolling body faults, respectively. The classification results obtained are entirely consistent with the actual conditions, with a recognition accuracy of 100% for the test set.

Similar to the bearing fault diagnosis experiment, 40 samples were collected for each state of the gear vibration signal and split into training and test sets in a 1:3 ratio. The test samples are organized into sequences representing the normal state (1–30), wear fault (31–60), crack fault (61–90), and missing tooth fault (91–120) conditions. The diagnostic results obtained using the proposed method are illustrated in Figure 6. Subplots (a) to (d) display the Marxist distances (MD1 to MD4) between the test sample sequences and the training samples for the normal state, wear fault, crack fault, and missing tooth fault, respectively.

**Figure 9:**
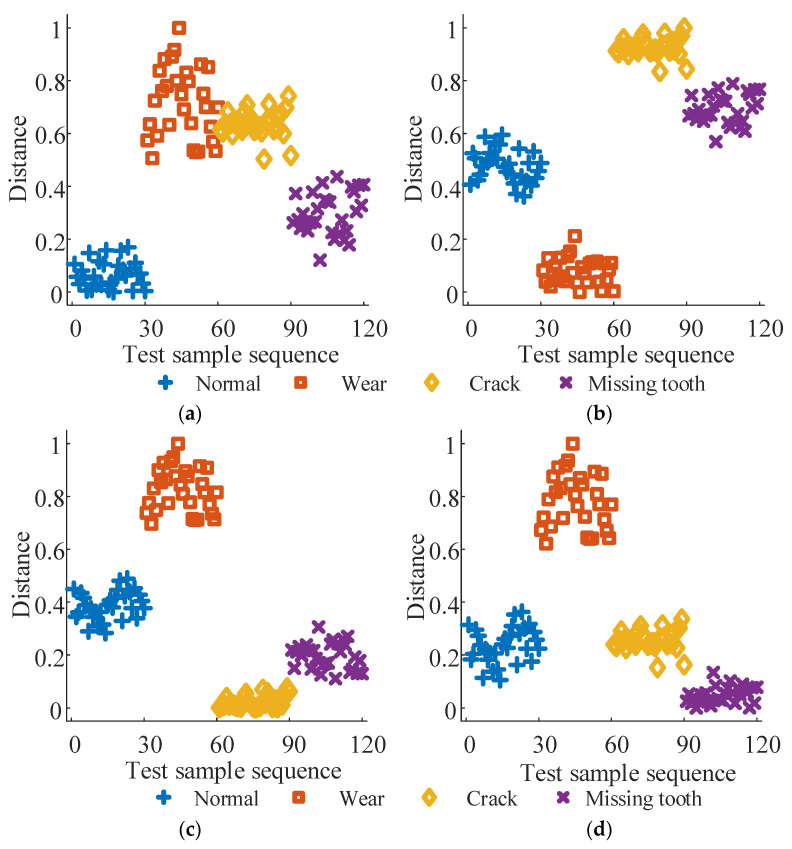
Gear fault diagnosis results. (**a**) MD1, (**b**) MD2, (**c**) MD3, and (**d**) MD4.

Based on Figure 9a–d, the test samples in sequences 1–22, 24–30, and 120 are identified as the normal state, with sequences 31–60 as wear fault, sequences 61–90 as crack fault, and sequences 14, 91–101, and 103–120 as missing tooth fault. These classifications align with the actual conditions. The average recognition accuracy of the test set is 98.33%, suggesting that the fault diagnosis method proposed in this paper is effective for diagnosing gear faults.

**Figure 10:**
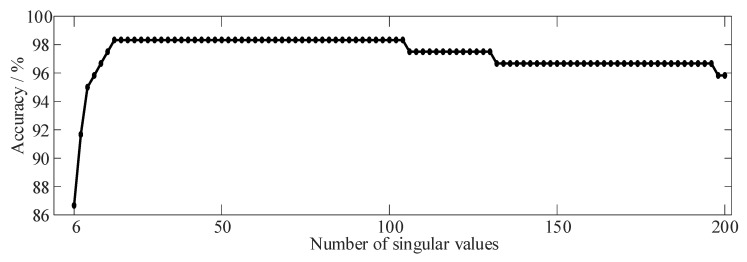
Variation curve of accuracy with the number of singular values.

In the proposed method, only the quantity of singular values needs to be manually specified. Varying the number of singular values utilized to construct the viewable representation results in fluctuations in the recognition rate of the proposed method. This indicates that the extraction of effective fault information is influenced by the number of singular values employed in constructing the graph signal. The correlation between the correct recognition rate and the number of singular values is depicted in Figure 10, derived from an extensive series of repeated experiments.

Analysis of Figure 10 reveals a clear trend: as the number of singular values used increases, the recognition accuracy of gear faults gradually improves. However, this upward trajectory peaks at 18 singular values, where recognition accuracy reaches its zenith and remains stable. Beyond this point, further increases in the number of singular values lead to a gradual decline in recognition accuracy. Surprisingly, even when utilizing the first 200 singular values, the accuracy rate remains notably high. This observation suggests a relatively loose selection criterion for the number of singular values in our method, with only the first 18 singular values utilized in the diagnosis results depicted in Figure 8.

Examining the characteristics of the singular value sequence, it becomes evident that the initial larger values primarily represent the signal’s main components. Consequently, when too few singular values are inputted into the VG algorithm, the resulting graph signal lacks sufficient fault information to comprehensively describe the gear’s fault characteristics. Conversely, an excessive number of singular values, particularly those representing noise components, can saturate the graph signal with limited fault information and potentially introduce interference, significantly increasing computational effort and time consumption. Therefore, constructing the viewable signal with only the initial larger singular values, which encapsulate the main feature information, ensures a comprehensive depiction of the gear’s fault state, mitigates noise interference, and minimizes computational resources and time consumption.

### 5.2. Comparison Experiment 1: Effect of Singular Value Topological Relationships on Diagnosis Results

To evaluate the superiority of the method proposed in this paper over traditional approaches that neglect the inter-relationship between singular values, we compare it with two such methods. Method I [7] entails constructing the original vibration signal into a Hankel matrix, followed by utilizing the complete sequence of singular values of the matrix as eigenvectors. In Method II [8], the vibration signal undergoes decomposition via the empirical mode decomposition (EMD) algorithm. Subsequently, the top k intrinsic mode functions (IMFs) with an accumulation percentage over 90% are selected to construct the spatial state matrix in the time–frequency domain, with the singular values of the state matrix serving as eigenvectors.

**Figure 11:**
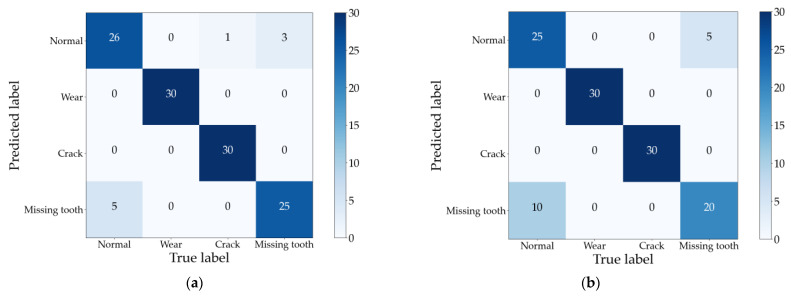
Diagnostic results of Method 1 and Method 2. (**a**) Method 1; (**b**)Method 2.

In this comparative experiment, we employ a gear fault dataset for testing, with a training-to-test sample ratio of 1:3. The Support Vector Machine (SVM) algorithm from the MATLAB classification toolbox is employed to train and classify the feature vectors constructed by the two traditional methods. The best classification among ten trials is selected as the result. Confusion matrices of the test sets for Method I and Method II are presented in Figure 11a and Figure 11b, respectively.

Observing Figure 11 reveals that the two traditional methods, which overlook the topological relationship of singular values, exhibit more misclassifications in their diagnosis results. Compared with the method proposed in this paper, their average accuracy is reduced by 5.83% and 10.83%, respectively. This deficiency stems from Method 1’s direct utilization of the singular value sequence as the feature vector and Method 2’s acquisition of a more refined singular value sequence via construction of the time–frequency domain state matrix. However, both approaches fail to fully harness the fault information embedded within the singular value sequence. In contrast, the method proposed in this paper incorporates the interdependence between singular values to extract hidden fault information, resulting in a more comprehensive understanding of faults.

**Figure 12:**
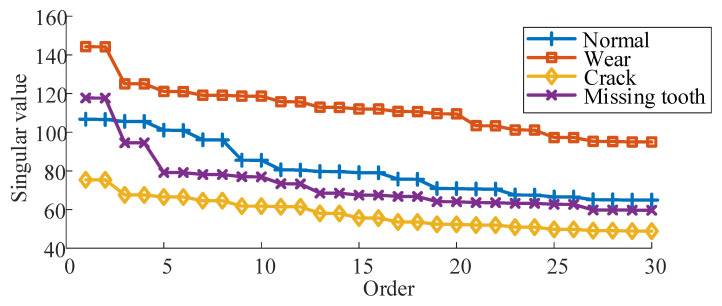
Singular value sequence of gear vibration signal in different states.

Furthermore, examination of Figure 8 and Figure 10 reveals that all three methods accurately identify wear faults and crack faults. However, misclassifications occur in diagnosing normal conditions and missing tooth faults. Notably, the misclassification results for these two fault types are reciprocal, as evident in Figure 10. This phenomenon is elucidated by Figure 12, which illustrates the first 30 singular values in the sequence of singular values of vibration signals for different gear fault states.

Figure 12 clearly demonstrates that the sequence of singular values for the normal state and missing tooth fault is closely aligned. This proximity contributes to the subpar classification performance when directly utilizing singular values as feature vectors to discern the normal state from the missing tooth fault. On the other hand, Method 2 relies on the decomposition quality of the EMD algorithm, which is more susceptible to noise. Consequently, the phenomenon of the impact signal amplitude decaying and increasing is less discernible amidst the influence of noise in the missing tooth fault samples. This circumstance results in mixed component information within the IMF components, thereby causing a significant number of misclassifications in the diagnosis outcomes of Method 2.

### 5.3. Comparison Experiment II: The Influence of the Composition Method on the Diagnosis Results

The inherent graph structure effortlessly captures the correlations between data elements by establishing edges between each pair of nodes. Leveraging this feature, fault information characterized by singular values can be further extracted by analyzing the graph signals with singular values as input using the graph signal processing (GSP) technique. However, since different methods of composition yield graph signals with distinct structures, Comparison II will focus on the performance of utilizing various composition methods in fault diagnosis.

**Figure 13:**
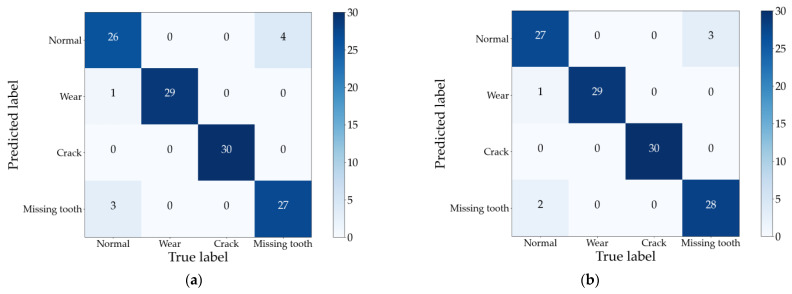
Diagnostic results of Method 3 and Method 4. (**a**) Method 3; (**b**) Method 4.

Numerous techniques exist for converting discrete sequences into graph signals. For instance, Wen et al. [22] proposed a method of constructing singular values as complete graphs to capture the fault characteristics of early failures in rolling bearings by encapsulating the long-term correlation between singular values. These graphs assume the existence of edge connections between all nodes. Conversely, the road graphs employed in the prior literature [13,14] represent a simpler class of graphs that only account for edge connections between neighboring nodes. In the forthcoming Comparison Experiment 2, these two composition methods will be juxtaposed with the Visibility Graph (VG) algorithm. In Method 3, singular values are depicted as complete graphs, with the weights of the adjacency matrix determined by the Euclidean distance between nodes. Conversely, in Method 4, singular values are depicted as road graphs. The remaining settings of both methods align with those outlined in this paper. The resulting confusion matrices of the test set are illustrated in Figure 13a and Figure 13b, respectively.

Upon comparing Figure 11 with Figure 13, it becomes evident that both Method 3 and Method 4 exhibit a modest improvement in diagnostic efficacy compared to the traditional method, which overlooks the topological relationship between singular values. However, misclassifications still predominantly occur in the normal state and missing tooth fault categories. This observation suggests that although Method 3 and Method 4 capture the interdependence between some singular values through the construction of complete graphs and road graphs, respectively, they fail to fully exploit the interdependence between singular values. This limitation arises from the simplistic edge connection rules defined and the complete disregard for differences among the singular values. Consequently, the diagnostic performance enhancement achieved by these methods is inferior to that of the approach presented in this paper.

### 5.4. Comparison Experiment 3: The Influence of Composition Elements on the Diagnosis Results

**Figure 14:**
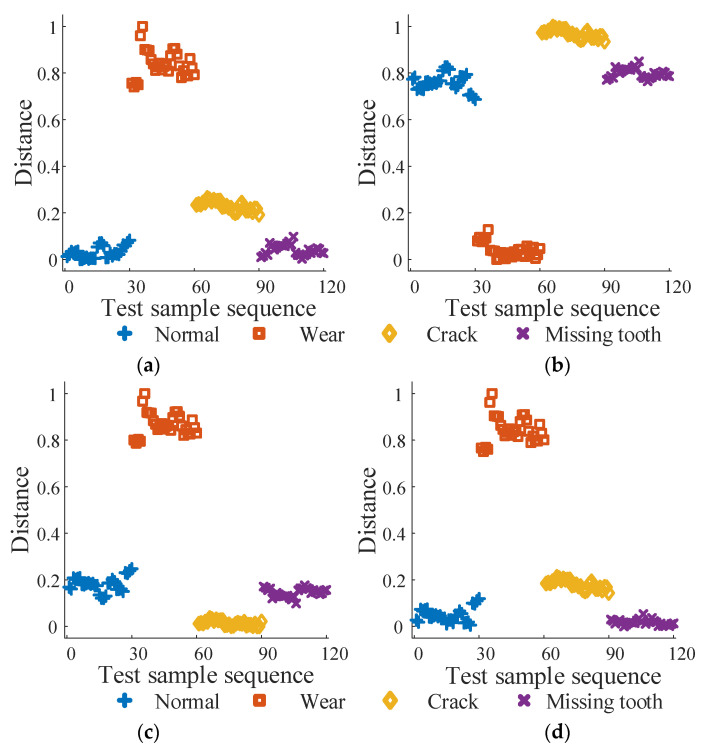
Diagnostic results of Method 5. (**a**) MD1, (**b**) MD2, (**c**) MD3, and (**d**) MD4.

The viewable signal has the capability to inherit certain characteristics of the original input sequence [23]. Consequently, employing different inputs will yield viewable signals that capture distinct fault information. Hence, Comparison Experiment 3 will scrutinize the impact of utilizing different inputs to construct the viewable signal on diagnosis outcomes. Moreover, extending the application of viewable signals to the domain of fault diagnosis, Chen et al. [24] introduced a method wherein the original vibration signal is directly employed as a viewable signal. The information entropy of the eigenvalue spectrum is then extracted as a fault feature for rolling bearing fault diagnosis. To this end, Method 5 was adopted as a comparative test in Comparison Experiment 3: the raw vibration signal of the gear served as the input for the Visibility Graph (VG) algorithm, while other settings remained consistent. The diagnosis results are delineated in Figure 14.

From Figure 14, it is apparent that Method 5 exhibits significant overlap in diagnosis results between the normal state and missing tooth fault categories, indicating the poorest diagnostic performance. Further analysis of this phenomenon reveals that the original vibration signal harbors substantial noise, wherein high-frequency noise components overshadow the main signal components. Consequently, a considerable amount of irrelevant noise information is incorporated into the constructed viewable signal, resulting in unsatisfactory diagnostic efficacy. This outcome starkly contrasts with methods employing singular values to construct the graph signal, underscoring the unsuitability of using the original vibration signal for constructing the viewable signal in fault diagnosis amidst a noisy background.

For ease of comparison, Table 1 presents the comparative results of the five methods alongside the approach proposed in this paper. Notably, the proposed diagnostic method achieves the highest recognition accuracy across the four gear fault states. In contrast to Method 1 and Method 2, this method leverages the graph structure to capture the topological relationship between singular values, effectively extracting fault information embedded within their interdependence, thus constructing more comprehensive and reliable feature parameters. Moreover, compared with Method 3 and Method 4, the proposed approach demonstrates superior retention of the structural features of the singular value sequence, thereby minimizing the loss of fault information concealed within the singular values. Finally, juxtaposed against Method 5, it is evident that constructing the viewable signal solely from main singular values containing feature information can circumvent noise component interference, enhancing robustness to noise and improving fault diagnosis accuracy.

**Figure 15:**
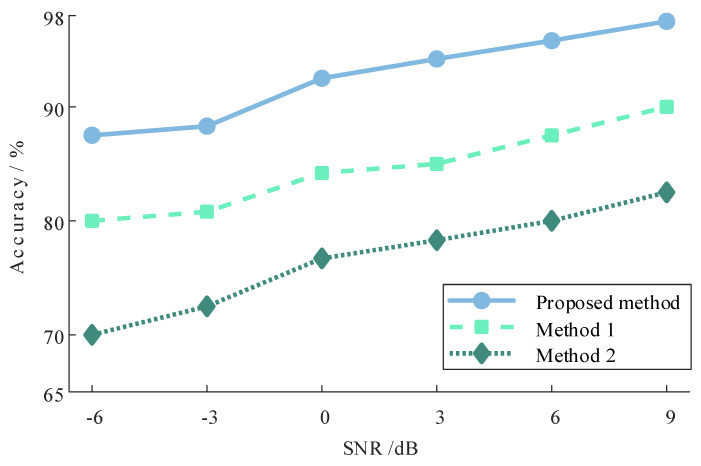
Comparison of diagnostic results of different SNRs.

To further validate the efficacy of the proposed method for diagnosing under conditions of strong noise, white noise is introduced into the gear signal to simulate the pervasive noise pollution prevalent in industrial environments. The outcomes of augmenting the signal with varying signal-to-noise ratio noises (−6 dB to 9 dB) for diagnosis, utilizing the method presented in this paper alongside the two compared methods, are depicted in Figure 15. It is noteworthy that the experimental setup maintains a consistent ratio of 1:3 for the training set to the test set.

Figure 15 illustrates a decline in accuracy across all three methods as the signal-to-noise ratio diminishes, exhibiting a notable reduction compared to pre-noise addition conditions. However, the proposed method consistently outperforms the comparison methods, maintaining a superior performance. Even at a signal-to-noise ratio of −6 dB, the proposed method achieves a recognition accuracy of 87.5%, surpassing the comparison methods by 7.5% and 17.5%, respectively. This underscores the robustness of the method presented in this paper, which retains its efficacy in ensuring accurate diagnosis even under conditions of pronounced noise.

## 6. Conclusions and Future Works

To accurately discern the operational state of wind turbine gearboxes, this study presents a novel gearbox fault diagnosis approach founded upon singular value decomposition (SVD) and graph Fourier transform (GFT), augmented by the integration of graph signal processing techniques. Addressing the limitations of conventional methods, which overlook the inter-relationships between singular values and consequently fail to extract comprehensive fault information from vibration signals, our method constructs viewable signals from the singular value sequences of gearbox vibration signals. Leveraging the inherent graph structure, the approach adeptly captures the topological relationships among singular value nodes, thereby uncovering hidden fault information.

Experimental evaluations conducted on two distinct datasets affirm the efficacy of the proposed method in gearbox fault diagnosis, achieving recognition accuracies exceeding 98% in both bearing and gear fault diagnoses. Comparative analyses against traditional methods underscore the superior accuracy in identifying fault types, validating the superiority of our proposed approach. Furthermore, under varying signal-to-noise ratios, our method consistently demonstrates higher recognition accuracy and robustness compared to traditional methods, affirming its efficacy and generalizability.

## Figures and Tables

**Table 1:** Fault classification results based on different methods.

Fault Type	Recognition Accuracy/%					
	Method 1	Method 2	Method 3	Method 4	Method 5	Proposed Method
Normal	86.67	83.33	86.67	90.00	60.00	96.67
Wear	100.00	100.00	96.67	96.67	100.00	100.00
Crack	100.00	100.00	100.00	100.00	100.00	100.00
Missing tooth	83.33	66.66	90.00	93.33	76.67	96.67
Average	92.50	87.50	93.33	95.00	84.17	98.33

## Data Availability

The data presented in this study are available upon request from the corresponding author. The data are not publicly available due to laboratory regulations.
